# Appendicio-ileal intertwining causing small bowel obstruction: a case report

**DOI:** 10.1097/MS9.0000000000005738

**Published:** 2026-10-08

**Authors:** Mulugeta W. Kedimu, Yonas T. Habtemariam, Dereje G. Andargie, Yoseph G. Kassie, Eden T. Habtemariam

**Affiliations:** a Department of Surgery, Debre tabor University, Debretabor, Ethiopia; b Department of Surgery, Bahir Dar University College of Medicine and Health science, Bahir Dar, Ethiopia; c Department of Surgery, Jimma University College of Public Health and Medical Sciences, Jimma, Ethiopia

**Keywords:** appendico-ileal intertwining, appendicitis, case report, knotting, strangulated small-bowel obstruction

## Abstract

**Introduction and importance:**

Although acute appendicitis is one of the most common causes of acute abdomen, appendico-ileal intertwining causing small-bowel obstruction is exceedingly rare. The diagnosis is almost always established intraoperatively. Early diagnosis and prompt surgical management are important, given the high risk of mortality.

**Case presentation:**

Here we report the case of a 34 year-old female patient with signs and symptoms of small bowel obstruction. Intraoperatively, the inflamed appendix entangled and obstructed the distal ileum. Appendectomy, ileal resection, and primary ileo-transverse anastomosis were performed. The patient had an uneventful recovery.

**Clinical discussion:**

Patients with appendico-ileal intertwining usually present with signs and symptoms similar to those of other causes of small-bowel obstruction. The management of appendico-ileal intertwining depends on the parts of the bowel involved and the level of strangulation. It ranges from simple appendectomy to resection of gangrenous bowel.

**Conclusion:**

Despite its rarity, appendico-ileal intertwining should be considered by the evaluating surgeon when encountering a case of intestinal obstruction. Delayed diagnosis or misdiagnosis can lead to bowel gangrene, sepsis, and ultimately death, especially in low-resource settings.

## Introduction

Small bowel obstruction (SBO) is defined as obstruction of the flow of small-bowel contents from the proximal to the distal segment^[^1^]^. The causes of SBO may be broadly classified as mechanical or functional. Among the mechanical causes, postoperative adhesions are the most common in developed countries, whereas small-bowel volvulus is the most common in Ethiopia^[^2,3^]^. Intestinal intertwining or knotting is the obstruction of an intestinal segment with a closed-loop phenomenon secondary to knotting of the mesentery^[^4^]^. It is one of the rarest causes of intestinal obstruction^[^5^]^.

HIGHLIGHTS

- Appendico-ileal intertwining is a condition in which the inflamed appendix wraps around a portion of the small intestine (ileum).
- Although appendico-ileal intertwining might be suspected before surgery, it is almost always diagnosed intraoperatively.
- Most patients have an uneventful course if appropriate surgery is performed early.
- As appendicitis is the most common cause of acute abdomen, every physician should be familiar with very rare and unusual presentations such as this case.

Acute appendicitis is one of the most common causes of acute abdomen^[^6^]^. The appendix, as a cause of mechanical intestinal obstruction, called appendicular knot or appendicular band syndrome, is uncommon and usually unsuspected. Although appendicular band syndrome was described as early as 1901, there are very few reports that provide a comprehensive review^[^7^]^. Early diagnosis and management are important, given the high risk of mortality^[^8^]^. Preoperative diagnosis of an appendico-ileal intertwining is challenging, and diagnosis is always made during surgery^[^7^]^. We present a case of small-bowel obstruction secondary to appendico-ileal intertwining, which was diagnosed during laparotomy. We present this case because of its rarity, its diagnostic challenge, and its potentially fatal outcome if diagnosis and intervention are delayed.

This case was reported in accordance with the SCARE Guidelines 2025 criteria^[^9^]^.

## Case presentation

A 34 year-old female patient from the North Western part of Ethiopia presented with central abdominal pain; nausea and bilious vomiting; and fever and chills. She also had abdominal distension and failure to pass feces and flatus for the past 2 days. She had no prior history of similar illness. She had no prior history of abdominal surgery. The patient had no significant past medical history.

On examination, the patient was tachycardic, febrile, and had low-normal blood pressure. Her abdomen was distended, and the hernia sites were free. There was generalized abdominal tenderness, which was more pronounced in the right lower quadrant. The abdomen had a hypertympanic percussion note, and bowel sounds were hyperactive. On per rectal examination, there was a small amount of formed stool. No gross abnormalities were detected in other systems.

On investigation, the complete blood count showed leukocytosis with a left shift in the neutrophil count. Other cell lines were within the normal range. Her blood group was O+. As the patient had clinical signs of peritonitis, no further tests were performed. She was resuscitated with intravenous fluids; gastrointestinal decompression was performed, she was kept NPO (nil per os), and started on broad-spectrum antibiotics. As she had no prior history of surgery or a clinically apparent hernia, small-bowel volvulus (being the predominant cause of obstruction in our setting) was considered the most likely differential diagnosis.

Subsequently, a decision was made to proceed with emergent laparotomy based on the clinical findings consistent with generalized peritonitis secondary to SBO. After written informed consent was obtained, the patient was taken to the operating room and placed in the supine position. Under general anesthesia, the abdomen was cleaned and draped. The peritoneal cavity was entered through a vertical midline incision. Intraoperatively, the small intestine was significantly distended, with approximately 300 mL of hemorrhagic fluid in the peritoneal cavity. The appendix was inflamed and wrapped around 20 cm of distal ileum (which was 5 cm away from the ileocecal valve), creating a closed-loop obstruction (Figs 1 and 2). The involved segment of ileum was frankly gangrenous, but there was no perforation. Considering the intraoperative findings, appendico-ileal intertwining was diagnosed. As the gangrenous distal 20 cm of ileum was close to the ileocecal valve, resection of the gangrenous distal ileum (Fig. 3) with appendectomy was performed. This was followed by a hand-sewn, double-layer, end-to-side ileotransverse anastomosis, and closure of the abdominal wall in layers. Postoperatively, the patient started sips on the second day and tolerated full feeding on the fifth day. She received broad-spectrum IV antibiotics for 6 days and analgesics, which she continued after discharge. She was discharged on postoperative day 6 with no complications. She had loose stools three times per day in her first week after discharge, which gradually resolved. She is currently 1 year postoperative and symptom-free.

**Figure 1.**
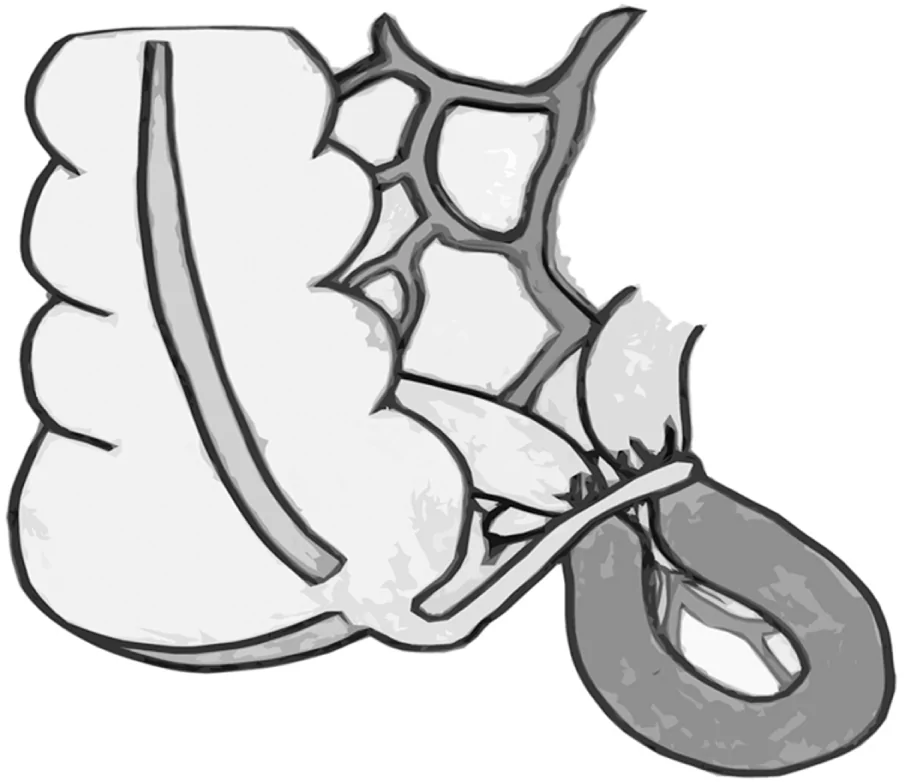
Schematic representation of the process of appendico-ileal intertwining – the appendix adheres to the terminal ileum, creating a potential space through which the small bowel herniates, leading to obstruction and strangulation.

**Figure 2.**
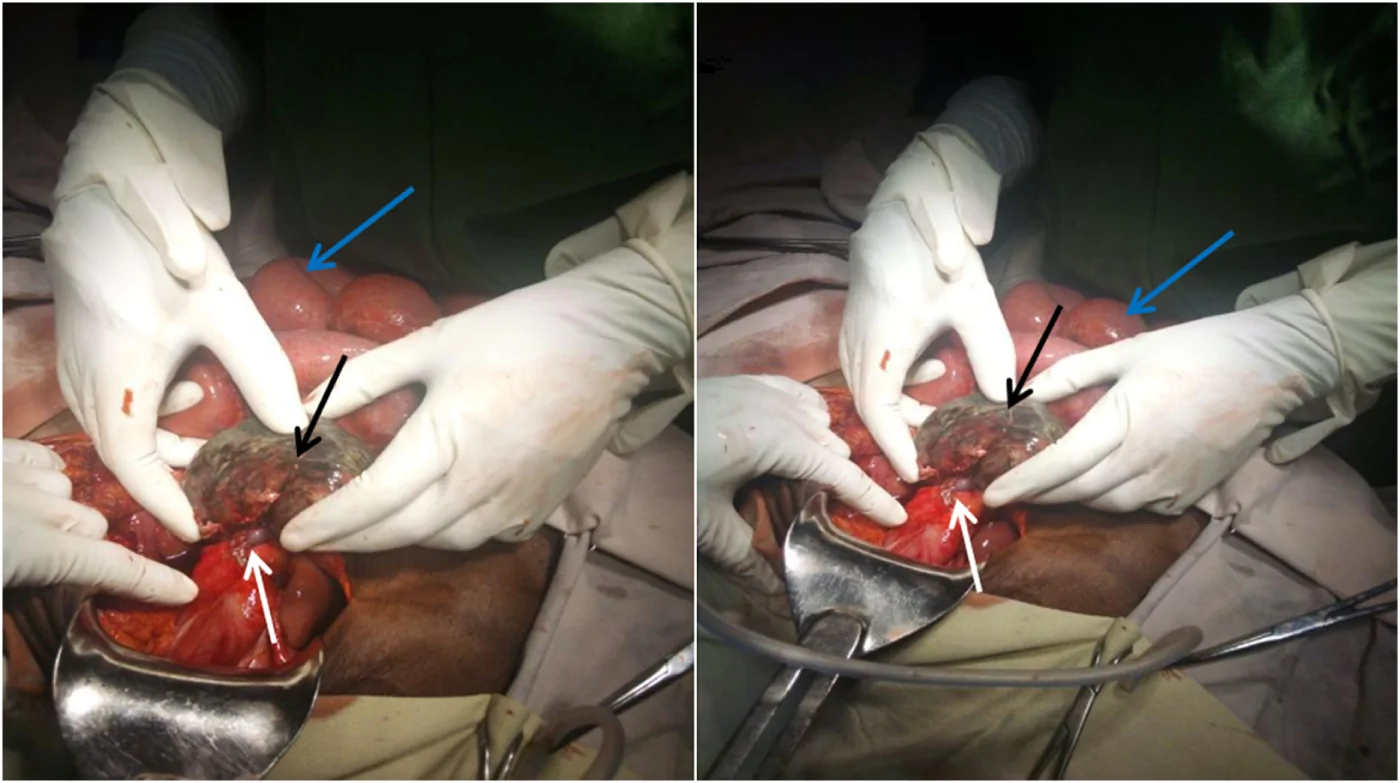
Intraoperative photographs show the frankly gangrenous ileum (black arrows) entrapped by an appendiceal tourniquet (white arrows). Blue arrows indicate dilated bowel loops proximal to the obstruction.

**Figure 3.**
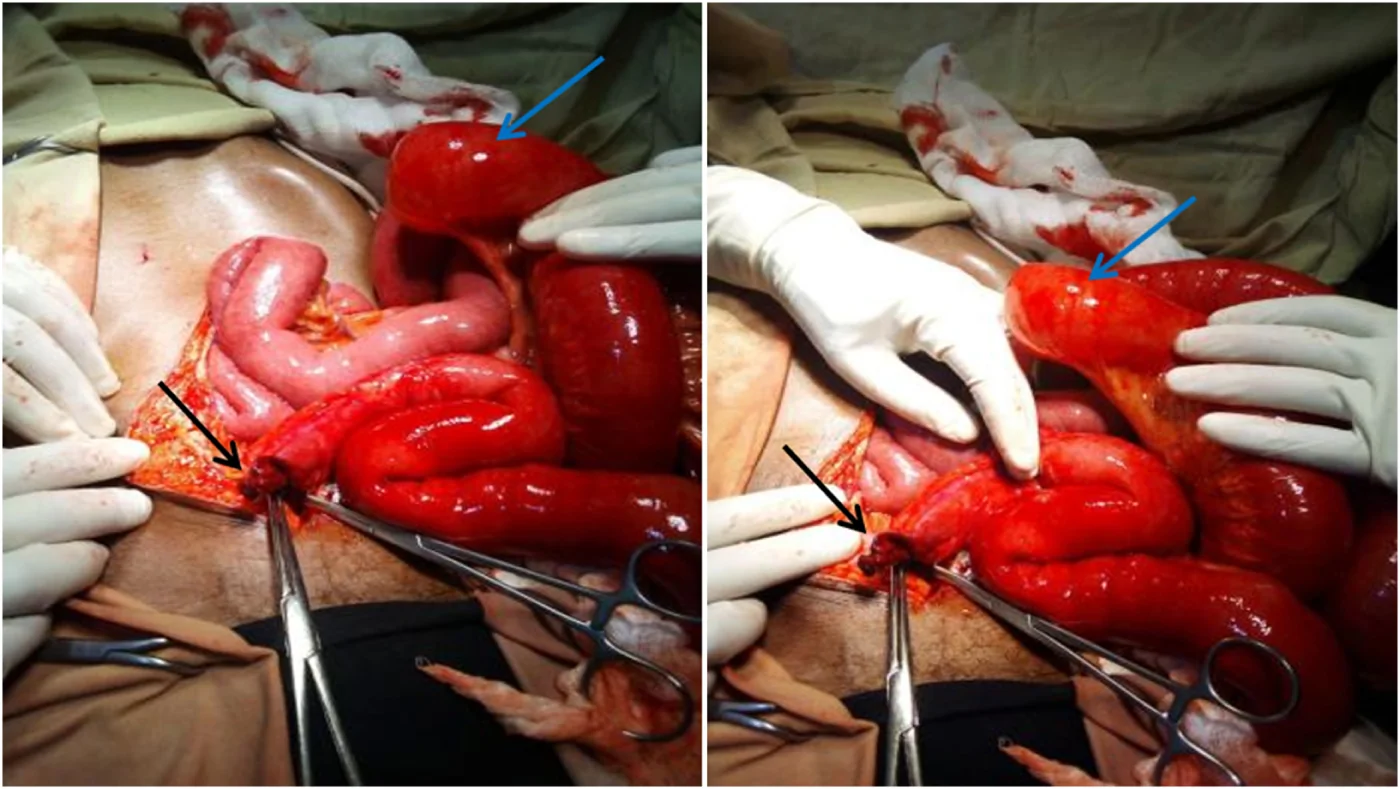
Intraoperative photographs showing the inflamed appendix (black arrows) after resection of the gangrenous segment of the ileum. Blue arrows point to the hyperemic, erythematous, and dilated ileum.

## Discussion

Acute appendicitis is the most common cause of an acute abdomen^[^7^]^. It occurs most often between the ages of 5 and 45, with a mean age of 28. The lifetime incidence is 8.6% for men and 6.7% for women^[^10^]^.

The natural history of untreated appendicitis includes the following complications: intra-abdominal abscess formation (3–4% in open appendectomy and 9–24% in laparoscopic appendectomy), prolonged ileus, enterocutaneous fistula, and small-bowel obstruction^[^10^]^. Acute appendicitis can cause intestinal obstruction either by causing small-bowel adynamic ileus or adhesions ^[^8^]^. Paralytic ileus secondary to appendicular inflammation is the most common cause of intestinal obstruction in patients with acute appendicitis, occurring in 1–5% of cases^[^11^]^. Three types of intestinal knotting have been described in the literature and can be ileosigmoid, ileoileal, or involve Meckel’s diverticulum or the appendix^[^4^]^. Mechanical small-bowel strangulation secondary to appendico-ileal knotting as a complication of acute appendicitis is uncommon^[^8^]^, as summarized in Table 1. Strangulation can result from the appendix wrapping around the base of a bowel loop. Alternatively, the inflamed appendix may adhere to the cecum, small intestine, or posterior peritoneum, allowing a portion of bowel to herniate through the gap. The former was the case in our patient. This is a rare occurrence, with only 10 other cases reported in the literature^[^11^]^. The first case of small-bowel strangulation secondary to appendicitis was described by Naumon in 1963^[^11^]^.

**Table 1:** Showing the types of Intestinal Knotting and reported cases in the indexed literature.

Intestinal knotting types	Number of reported cases	Frequency of occurrence	Notes
Ileosigmoid knot	>900 reported cases	Commonest type	Common in Africa and Asia.
			Highest risk of strangulation
Ileoileal knot	24 reported cases	Rare	Common in Africa, India, Asia and East Europe.
			Has the best prognosis
Appendico-ileal knot	10 reported cases	Rare	Diagnosed during laparotomy; one reported death (due to delayed surgery)

References: Ileosigmoid knot^[^19^]^; Ileoileal knot^[^5–29^]^; Appendicio-ileal knot^[^7,8,11,12,14–18^]^.

Appendico-ileal intertwining generally presents in one of two ways: the more common presentation is a predominant picture of acute intestinal obstruction. The second presentation is predominant features of appendicitis accompanied by some evidence of intestinal obstruction^[^12^]^. Our patient also had predominant features of acute intestinal obstruction. Preoperative diagnosis of appendico-ileal intertwining is very challenging^[^7^]^. Similar to another case reported in the southwestern part of Ethiopia, we also could not diagnose the appendico-ileal intertwining preoperatively^[^13^]^.

Imaging studies, such as CT scans of the abdomen, might give clues to the preoperative diagnosis, but this is usually difficult in resource-limited settings such as ours. As patients usually present with an acute abdomen, emergent exploration is indicated in almost all cases^[^13,14^]^. The management of appendico-ileal intertwining varies based on the parts of the bowel involved and the level of strangulation. It ranges from appendectomy to resection of the gangrenous bowel segment^[^13^]^. In our patient, the entrapped gangrenous ileum was located 5 cm proximal to the ileocecal valve. Consequently, preservation of the ileocecal junction was not feasible, and resection of the gangrenous bowel with appendectomy and ileotransverse anastomosis was performed. Similar to our case, three of the reported cases had gangrenous small bowel necessitating small bowel resection and anastomosis^[^13,15,16^]^. Although these reports also described gangrenous bowel requiring resection, the extent and distal location of gangrene in our patient necessitated a more complex reconstruction. Unlike our case, the entangled small bowel was viable in six reported cases, and appendectomy alone was sufficient^[^7,11,12,14,17,18^]^. Despite the severity of the disease, the patient experienced an uneventful postoperative course and was symptom-free at 1-year follow-up, highlighting that favorable long-term outcomes can be achieved with timely surgical intervention. As this is a single case report, the conclusions may be inherently limited; however, this case gives insight into the diagnosis and surgical management of appendico-ileal intertwining in resource-limited settings. The limitations portrayed here may be relevant to surgeons working in similar resource-limited situations.

## Conclusion

The most common causes of SBO are postoperative adhesions and hernias. Despite its rarity, appendico-ileal intertwining should be considered by the evaluating surgeon when encountering a case of intestinal obstruction. This is especially true in low-resource settings where investigative modalities are not readily available. Late diagnosis or misdiagnosis can lead to bowel gangrene, sepsis, and ultimately death.

## Data Availability

Data available on request from the authors.

## Research registration unique identifying number (UIN)

Name of the registry: Researchregistry.com. Unique identifying number or registration ID: researchregistry11298.
